# Induction of PLSCR1 in a STING/IRF3-Dependent Manner upon Vector Transfection in Ovarian Epithelial Cells

**DOI:** 10.1371/journal.pone.0117464

**Published:** 2015-02-06

**Authors:** Karthik M. Kodigepalli, Meera Nanjundan

**Affiliations:** Department of Cell Biology, Microbiology, and Molecular Biology, University of South Florida, Tampa, Florida, 33620, United States of America; SRI International, UNITED STATES

## Abstract

Toll-like receptors (TLRs) are the primary sensors of the innate immune system that recognize pathogenic nucleic acids including double-stranded plasmid DNA (dsDNA). TLR signaling activates multiple pathways including IRF3 which is involved in transcriptional induction of inflammatory cytokines (i.e. interferons (IFNs)). Phospholipid scramblase 1, PLSCR1, is a highly inducible IFN-regulated gene mediating anti-viral properties of IFNs. Herein, we report a novel finding that dsDNA transfection in T80 immortalized normal ovarian surface epithelial cell line leads to a marked increase in PLSCR1 mRNA and protein. We also noted a comparable response in primary mammary epithelial cells (HMECs). Similar to IFN-2α treated cells, *de novo* synthesized PLSCR1 was localized predominantly to the plasma membrane. dsDNA transfection, in T80 and HMEC cells, led to activation of MAPK and IRF3. Although inhibition of MAPK (using U0126) did not modulate PLSCR1 mRNA and protein, IRF3 knockdown (using siRNA) significantly ablated the PLSCR1 induction. In prior studies, the activation of IRF3 was shown to be mediated by cGAS-STING pathway. To investigate the contribution of STING to PLSCR1 induction, we utilized siRNA to reduce STING expression and observed that PLSCR1 protein was markedly reduced. In contrast to normal T80/HMECs, the phosphorylation of IRF3 as well as induction of STING and PLSCR1 were absent in ovarian cancer cells (serous, clear cell, and endometrioid) suggesting that the STING/IRF3 pathway may be dysregulated in these cancer cells. However, we also noted induction of different TLR and IFN mRNAs between the T80 and HEY (serous epithelial ovarian carcinoma) cell lines upon dsDNA transfection. Collectively, these results indicate that the STING/IRF3 pathway, activated following dsDNA transfection, contributes to upregulation of PLSCR1 in ovarian epithelial cells.

## Introduction

Plasmid DNA transfection is one of the most commonly used tools in biology to achieve exogenous expression of specific proteins of interest in mammalian cells. Entry of plasmid DNA harboring the gene of interest can be facilitated by cationic lipid-based transfection reagents [1]. Microarray gene expression studies suggest that plasmid transfection results in induction of genes associated with regulating primary immune responses upon viral/foreign DNA entry including interferons (IFNs) and other inflammatory cytokines [2]. This event is similar to cellular recognition of foreign nucleic acids by Toll-like Receptors (TLRs) which can be subclassified into two major groups. TLR1, 2, 4, 5, 6, and 10 are plasma membrane localized and are involved in the recognition of pathogenic protein components including viral envelope proteins or bacterial wall proteins [3]. TLR3, 7, 8, and 9 are localized to endosomal compartments from the endoplasmic reticulum and are involved in sensing pathogenic (viral/bacterial) and non-pathogenic (plasmid DNA) foreign nucleic acids [4–6]. Activation of TLRs leads to activation of downstream signaling mediators including PI3K [7], MAPK [8,9], and interferon regulatory factors (i.e. IRF3/7) which are responsible for regulating expression of specific IFN-dependent genes [10,11]. Other recently identified cytosolic sensing pathways include the cGAS-cGAMP-STING pathway [12,13].

Phospholipid scramblase 1 (PLSCR1), located at 3q23, is a well-established target of IFN signaling and an important mediator of anti-viral functions of IFNs [14–19]. PLSCR1 is transcriptionally regulated by IFN via a signaling pathway involving activation of PKC-δ, JNK, and STAT1 [20]. Interestingly, PLSCR1 can regulate TLR9 signaling pathway and the subsequent IFN production in plasmacytoid dendritic cells [21]. Although primarily localized to plasma membrane, PLSCR1 has also been detected in the nucleus, endoplasmic reticulum, Golgi, and endosomal compartments under specific conditions (i.e. IFN and 2-bromopalmitate treatment) [22–24]. In addition to its anti-viral function, PLSCR1 appears to be implicated in cancer development and cellular responses to chemotherapeutic agents [25–30].

Herein, we report that transfection of empty plasmid (dsDNA) in LTAg/hTERT immortalized normal ovarian surface epithelial cells (T80) and primary mammary epithelial cells (HMEC) leads to a marked induction of endogenous PLSCR1 expression. To identify the mechanisms leading to dsDNA-mediated PLSCR1 induction, we assessed the activation of molecules downstream in the TLR signaling cascade including STAT3, JNK, PKC-δ, and IRF3 in dsDNA-transfected T80 cells. We observed a marked activation of IRF3 as well as induction of Type 1 IFNs (specifically, IFN-α and IFN-β). In addition, we detected a significant mRNA induction of TLR4 and TLR9. Strikingly, IRF3 knockdown (via siRNA) led to a marked reduction in PLSCR1 expression implicating IRF3 in the transcriptional regulation of PLSCR1. Thus, we next assessed upstream pathways which are known to activate IRF3 including STING. Strikingly, knockdown of STING dramatically reduced PLSCR1 protein. In contrast to T80 and HMEC cells, we did not observe increases in PLSCR1 expression (or p-IRF3) in ovarian cancer cell lines, despite similar transfection efficiencies between T80 and the ovarian cancer cell lines (HEY, TOV21G, and TOV112D). Collectively, these studies suggest that activation of the STING/IRF3 cascade may be responsible for PLSCR1 induction in response to dsDNA transfection in normal epithelial cells. The significance of this observation with respect to ovarian cancer development remains to be investigated.

## Materials and Methods

### Materials

pcDNA3 and pGL3-basic vectors were kindly provided by Dr. Peter J. Sims (University of Rochester, NY). pBABE-puro retroviral vector was obtained from Dr. Gordon B. Mills (MD Anderson Cancer Center, TX). pQCXIN retroviral vector was purchased from Clontech (Mountain View, CA). PLSCR1 (1E9) mouse monoclonal antibody (1:500, sc-59645) was obtained from Santa Cruz Biotechnology (Dallas, TX). PLSCR1 (4D2) mouse monoclonal antibody [31] was a kind gift from Dr. Peter J. Sims (University of Rochester, NY) and Dr. Marc Benhamou (University Paris Diderot, FR). Phospho-SAPK/JNK mouse monoclonal (1:500, #9255), phospho-STAT3 rabbit polyclonal (1:1000, #9145), phospho-p44/42 MAPK (pERK) rabbit polyclonal (1:1000, #9101), phospho-AKT (S473) rabbit polyclonal (1:1000, #4060), phospho-IRF3 (S396) rabbit monoclonal (1:500, #4947), p44/42 MAPK rabbit polyclonal (1:1000, #4695), AKT rabbit polyclonal (1:1000, #4685), STAT3 rabbit polyclonal (1:1000. #4904), IRF-3 rabbit monoclonal (1:1000, #11904), phospho-PKCδ/θ (1:500, #9376), phospho-STAT1 rabbit monoclonal (1:1000, #8826), STAT1 rabbit polyclonal (1:1000, #9172), STING rabbit monoclonal (#13647), PKCδ rabbit monoclonal (1:1000, #9616), and pan-actin rabbit polyclonal (1:1000, #4968) antibodies were obtained from Cell Signaling Technology (Danvers, MA). The MEK1/2 selective inhibitor, U0126 (#9903), was obtained from Cell Signaling Technology (MA). IFN-2α (#JM-4595-100) was obtained from MBL International (Des Plaines, IL).

### Cell culture and treatments

LTAg/hTERT immortalized normal ovarian surface epithelial cells (T80) as well as the HEY ovarian carcinoma cells (kindly provided by Dr. Gordon Mills [32,33], MD Anderson Cancer Center, TX) were cultured in RPMI 1640 media supplemented with 8% fetal bovine serum and penicillin/streptomycin. The clear cell ovarian carcinoma cell line, TOV21G, was kindly provided by Dr. Jonathan Lancaster (Moffitt Cancer Center, FL) [34,35]. The endometrioid ovarian carcinoma cell line, TOV112D, was obtained from ATCC (#CRL-11731, VA). Both TOV112D and TOV21G cells were maintained in MCDB131:Medium 199 (1:1 ratio) supplemented with 8% FBS and penicillin/streptomycin. The cell lines were subcultured by trypsinization using 0.25% Trypsin-EDTA and passaged at a 1:5 dilution. The human primary mammary epithelial cells (HMEC) were obtained from ATCC (PCS-600-010) and were cultured in Mammary Epithelial Cell Basal Medium (#PCS-600-030) supplemented with components of the Mammary Epithelial Growth Kit (#PCS-600-040); these cells were subcultured as described above using 0.05% Trypsin-EDTA. All cell lines utilized herein were STR profiled (Genetica Laboratories, NC) and mycoplasma tested to be negative.

T80, HEY, TOV21G, and TOV112D cells were seeded at 250,000 cells per well in 6-well plates or 35mm culture dishes. Cells were allowed to attach overnight prior to treatments or transfections. Cells were treated with 3000 IU/ml interferon-2α (IFN-2α) prior to assessment of PLSCR1 induction. For inhibition of the MAPK signaling pathway, pre-treatment with U0126 (10 μM) or an equal volume of DMSO was performed for 2 hours.

### Plasmid transfection

T80, HEY, TOV21G, and TOV112D were seeded at 250,000 cells/well in a 6-well plate or 35mm dishes (onto glass coverslips for immunofluorescence (as described below)) whereas HMEC cells were seeded at a density of 52,000 cells per well in 24-well plates (onto glass coverslips for immunofluorescence (as described below)). Following overnight adherence, cells were transfected at 1 μg plasmid per well using Fugene HD (Promega, WI) as previously described [36]. For the studies involving U0126, cells were first pre-treated with 10 μM U0126 containing media for at least 2 hours prior to plasmid transfection. Six hours post-transfection, cells were overlayed with 2 ml of complete growth media containing U0126.

### siRNA transfection

T80 cells were seeded at 250,000 cells/well in 6-well plates or 35mm dishes. Following overnight attachment, cells were transfected with one round of the following ON-TARGETplus siRNAs (Dharmacon, Lafayette, CO): non-targeting control siRNA (D-001810), PLSCR1 (L-003729-00), TLR4 (L-008088-01), TLR9 (L-004066-00), IRF3 (L-006875-00), STAT1 (L-003543-00), STAT3 (L-003544-00), and STING (L-024333-02). Dharmafect I was used as the transfection reagent (Dharmacon). The siRNA transfections were performed as described previously [37].

### Protein isolation, SDS-PAGE, and western analyses

Total cell lysates were prepared by incubating cells in RPPA lysis buffer (1% Triton X-100, 50 mM HEPES, 150 mM NaCl, 1 mM MgCl_2,_ 1 mM EGTA, 10% glycerol, and protease inhibitor cocktail) or RIPA lysis buffer (50 mM Tris-HCl [pH 7.4], 150 mM NaCl, 0.5% sodium deoxycholate, 0.1% SDS and 1% NP-40) for 1 h at 4°C followed by centrifugation at 14,000 rpm for 10 minutes. Total protein was normalized to at least 1 mg/ml, resolved on 10% SDS-PAGE gels, and transferred to polyvinylidene fluoride (PVDF) membranes. Western analyses were performed as described previously utilizing the antibodies mentioned above [37].

### Quantitative PCR

Total RNA was extracted from the T80 and HEY cells using the RNeasy mini kit (Qiagen, CA). Quantitative PCR was performed using One-Step PCR Taqman master mix and the One-Step-Plus detection system (Applied Biosystems, CA). Probes/primers specific for PLSCR1 (Hs00275514_m1), IFN-α (Hs00265051_s1), IFN-β (Hs01077958_s1), IFN-γ (Hs00989291_m1), TLR4 (Hs00152939_m1), and TLR9 (Hs00370913_s1) were obtained from Applied Biosystems (Assays-on-Demand). β-actin (#401846) was used as the endogenous control. PCR conditions and data analysis were conducted as previously described [37].

### Immunocytochemistry

T80 cells were seeded onto glass coverslips in 6-well plates or 35mm dishes. Following overnight attachment, cells were treated with IFN-2α or transfected with pcDNA3 vector as described above. Staining was performed using methods as described previously [28]. Briefly, cells were fixed with 4% formaldehyde for 30 minutes followed by blocking for 1 hour in PBS containing 0.1% Triton-X-100 with 5% goat serum. Cells were next incubated overnight at 4°C using a 1:500 dilution of PLSCR1 (4D2) antibody (prepared in PBS containing 0.1% Triton-X-100 and 1% goat serum). Next, the cells were washed with PBS and then incubated in Alexafluor-488 conjugated goat anti-mouse secondary antibody (1:500, #A11029, Invitrogen, NY) for 1 hour at room temperature. After three PBS washes, coverslips were mounted onto glass slides together with mounting media containing DAPI. Slides were then viewed and imaged using a Perkin Elmer UltraVIEW Confocal spinning disc microscope (Perkin Elmer Corporation, Waltham, MA). For the secondary antibody only control, cells were fixed, blocked, and incubated directly in secondary Alexafluor-488 conjugated antibody.

### Annexin V-PI apoptosis assay

Annexin V and PI staining was performed as described previously [38] using a kit obtained from Calbiochem (Rockland, MA). Forty-eight hours post-transfection with pcDNA3, supernatant (containing detached T80 cells) was collected and combined with the adherent cells (which were trypsinized). Stained cells were then analyzed via flow cytometry (Karoly Szekeres, College of Medicine, University of South Florida, Tampa, FL).

### Assessment of transfection efficiency

Cells were plated in 6-well plates (T80, HEY, TOV21G, and TOV112D) or 24-well plates (HMEC) at a density of 250,000 or 52,000 cells per well, respectively with or without coverslips. Following adherence, cells were transfected with 1 μg of pEGFP-C1 plasmid (Clontech, CA). Forty-eight hours post-transfection, cells were then processed for analysis via flow cytometry or via immunofluorescence microscopy as described above. Cells positive for GFP were quantified and the percentage of GFP positive cells was calculated to determine the transfection efficiency.

### Statistical analyses

Graphical representation of data was generated using the averages of all the independent replicates with error bars representing the standard deviations. The number of independent replicates is specified in the Figure Legends.

## Results

### dsDNA transfection leads to induction of PLSCR1 in T80 ovarian surface epithelial cells and HMEC epithelial mammary cells

**Fig 1:**
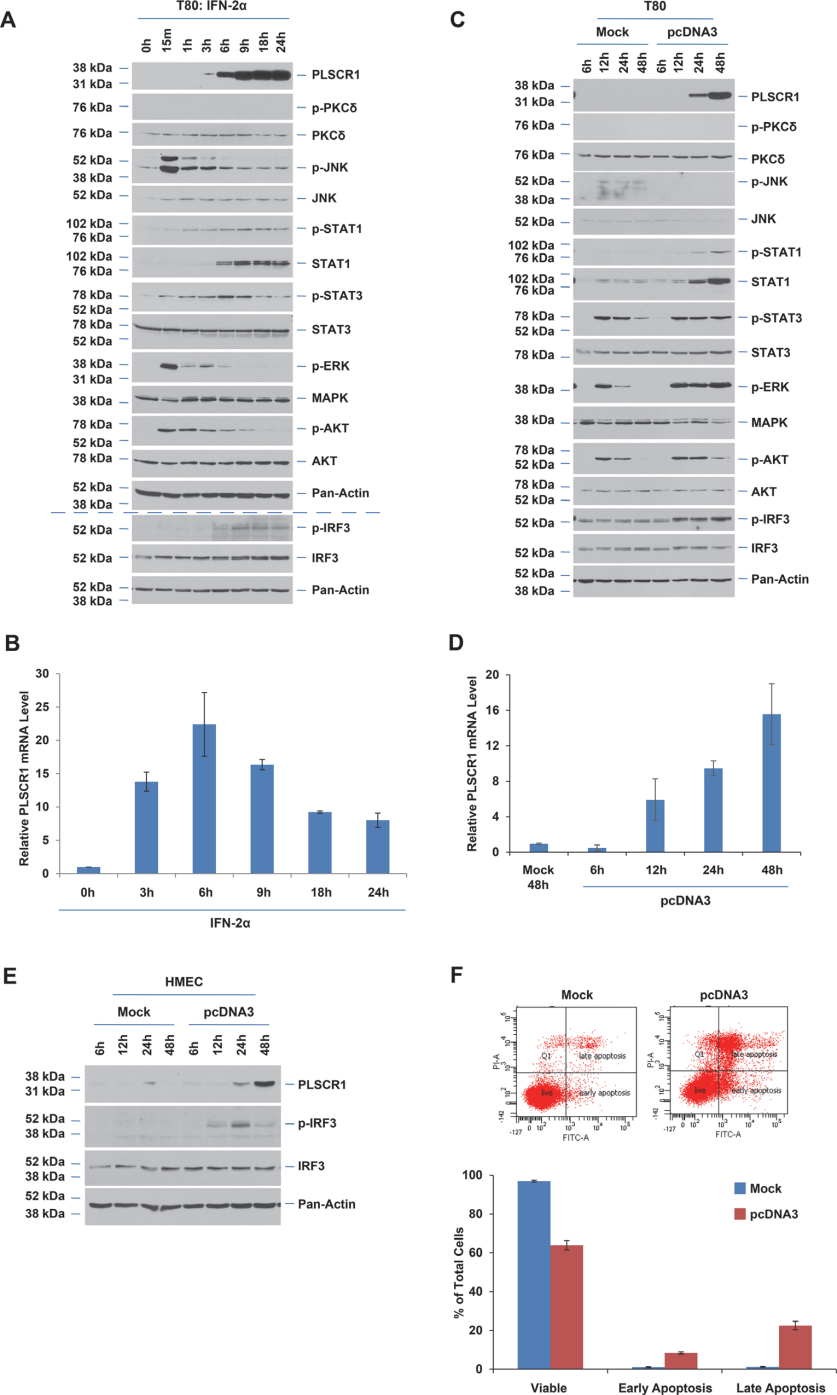
IFN and empty plasmid transfection induce PLSCR1 mRNA and protein. (**A**) T80 cells were treated with 3000 IU/ml IFN-2α from 15 minutes up to 24 hours. Cell lysates were analyzed by western blotting with the indicated antibodies (n = 3). (**B**) Total RNA was isolated from cells treated as described in (A). PLSCR1 mRNA levels, detected via real-time PCR, are presented (n = 3). (**C**) T80 cells were transfected with empty pcDNA3 plasmid (“pcDNA3”) or transfection reagent only (“mock”). Cell lysates were harvested from 6 up to 48 hours post-transfection and analyzed via western blotting with the indicated antibodies (n = 3). (**D**) Total RNA was isolated from cells treated as described in (**C**). PLSCR1 mRNA levels, detected via real-time PCR, are presented (n = 4). (**E**) HMEC cells were treated similarly as described for T80 cells (C) and cell lysates were then analyzed by western blotting with the indicated antibodies (n = 2). (**F**) Annexin V-PI staining was performed in 48 hours mock or pcDNA3 transfected cells (n = 3).

In order to determine whether PLSCR1 expression is modulated by dsDNA transfection, we selected an immortalized ovarian epithelial cell line (T80) and performed transfection with the pcDNA3 plasmid (dsDNA, 5.4 kb). We compared this response to that of IFN-2α since plasmid transfection can lead to induction of IFN-inducible genes [39]. As shown in Fig. 1A, T80 cells were treated with 3000 IU/ml IFN-2α from 15 minutes to 24 hours. However, similar to our previous results in the HEY ovarian cancer cell line [28], we observed that PLSCR1 protein was markedly induced from 6 until 24 hours (basal PLSCR1 protein level is very low). Previous studies have reported that PLSCR1 induction upon IFN-2α stimulation is mediated by protein kinase C-δ (PKC-δ), c-Jun N-terminal kinase (JNK), and signal transducer and activator of transcription (STAT1) activation [20]. Although we identified that JNK (15 minutes), STAT1 (1–3 hours), and STAT3 (6–9 hours) are activated following IFN-2α stimulation in the T80 cells, PKC-δ activation was undetectable under our conditions (Fig. 1A). To determine whether this could be due to sample preparation and lysis conditions, we isolated protein lysates using RPPA and RIPA buffer to determine whether the detection of p-JNK and p-PKC-δ could be improved. However, as shown in Fig. A-A in S1 File, we did not observe any improvement in detection with RIPA buffer. Furthermore, since we detected an increase in total STAT1 protein levels from 6 hours to 24 hours, the increase in p-STAT1 at these time points is likely due to the increase in total STAT1 protein. Additionally, we detected activation of MAPK as well as AKT (Fig. 1A). Activation of these pathways was transient in nature (rapidly increasing at 15 minutes followed by a rapid decay at 1 hour). In order to determine whether the increase in PLSCR1 protein upon IFN-2α stimulation was due to increased transcriptional activation, we next performed quantitative PCR utilizing specific probes/primers targeting PLSCR1. We treated T80 cells with 3000 IU/ml IFN-2α from 3 to 24 hours. As shown in Fig. 1B, the mRNA levels of PLSCR1 markedly increased at 6 hours followed by a reduction until 24 hours.

As shown in Fig. 1C, lysates from T80 cells transfected with pcDNA3 were collected between 6 to 48 hours post-transfection and compared to mock transfected cells. Western analysis demonstrates a marked induction of PLSCR1 protein starting at 24 until 48 hours post-transfection. In comparison, there was no induction of PLSCR1 protein in mock transfected cells. In contrast to IFN-2α treatment, we did not detect activation of JNK or PKC-δ upon plasmid transfection (relative to mock). The use of RIPA buffer did not improve detection of these phosphorylated proteins (Fig. A-B in S1 File). Moreover, we noted an increase in p-STAT1 but total STAT1 protein was also increased at 24 and 48 hours post-dsDNA transfection. For MAPK and AKT activation, it appeared that the transfection with dsDNA led to a dramatic sustained activation of MAPK while AKT activation was more subtle (Fig. 1C). Since TLR and cGAS-STING signaling pathways are activated following cellular recognition of foreign DNA [12,13,39,40], we next assessed the activation status of IRF3, a common downstream target of these pathways. We observed a marked increase in phosphorylated IRF3 upon dsDNA transfection (relative to mock) suggesting that TLR signaling may be activated upon cellular entry of dsDNA (Fig. 1C). Similar to dsDNA transfection, activation of IRF3 was also detectable upon IFN-2α treatment starting at 6 hours (Fig. 1A). To determine whether induction of PLSCR1 was transcriptionally mediated, we performed real-time PCR using dsDNA transfected T80 cells relative to mock transfection (48 hours) (Fig. 1D). Indeed, the PLSCR1 mRNA significantly increased from 12 until 48 hours post-transfection. To assess whether induction of PLSCR1 following dsDNA transfection could occur in other epithelial cell types, we assessed the effects of dsDNA transfection in primary mammary epithelial cells (HMECs). As shown in Fig. 1E, we observed that PLSCR1 protein was induced markedly from 24 to 48 hours post-transfection (relative to mock). We also noted that IRF3 was activated upon dsDNA transfection in HMECs. Collectively, these results indicate that entry of dsDNA into epithelial cells leads to phosphorylated IRF3, a downstream mediator of the TLR signaling cascade, as well as an upregulation of PLSCR1 mRNA and protein.

We next assessed whether cellular viability was altered following dsDNA transfection. As shown in Fig. 1F, we noted a ∼30% increase in dead cells (early and late apoptotic populations) upon dsDNA transfection compared to mock transfected cells. These results indicate that plasmid transfection can dramatically alter cellular survival in T80 cells.

### dsDNA transfection leads to plasma membrane localization of *de novo* synthesized PLSCR1 in T80 ovarian surface epithelial cells

**Fig 2:**
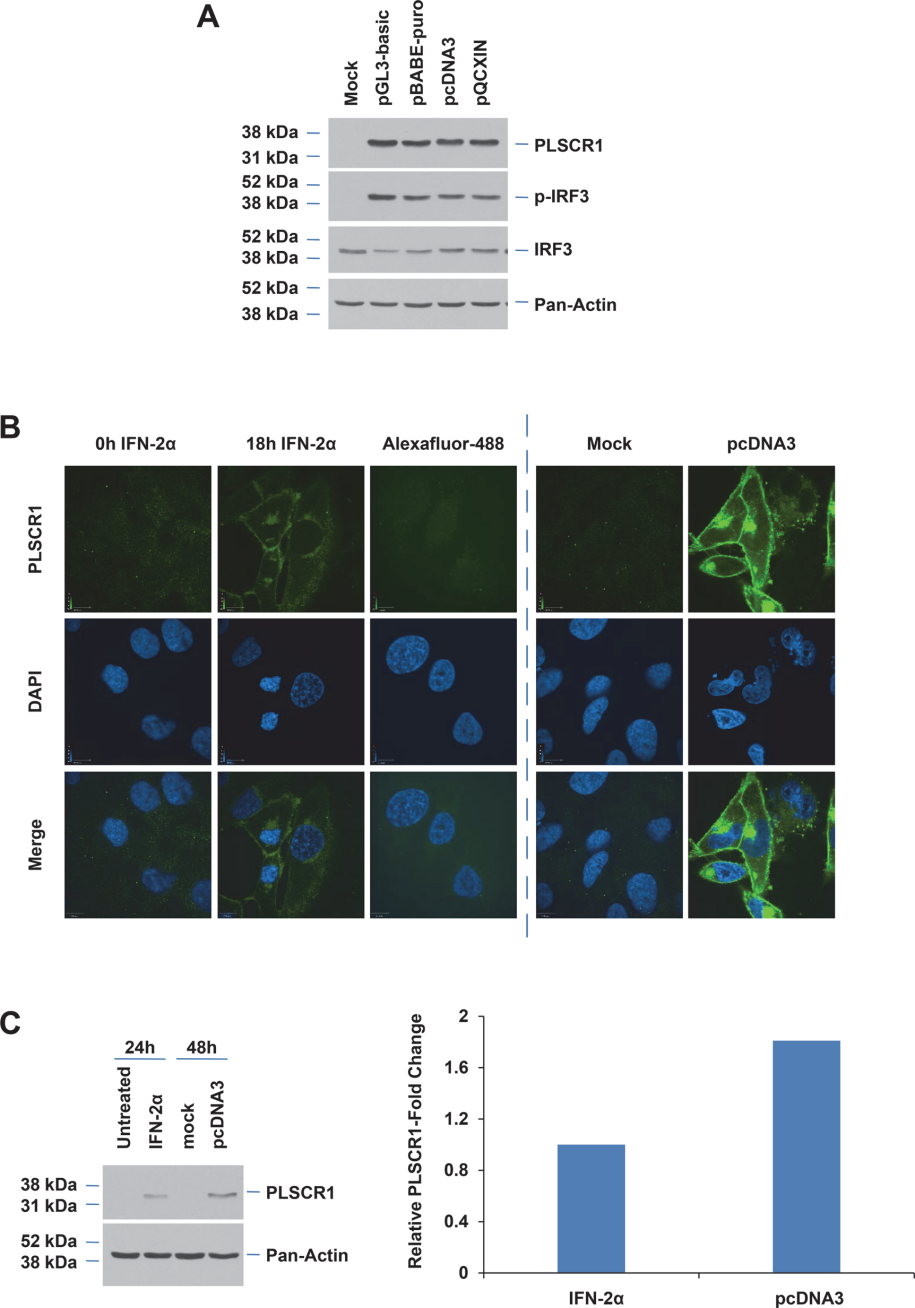
Subcellular localization of PLSCR1 following IFN treatment and dsDNA transfection. (**A**) T80 cells, transfected with empty pGL3-basic, pBABE-puro, pcDNA3, pQCXIN vectors, or transfection reagent only (“Mock”) were analyzed via western blotting with the specified antibodies (n = 3). (**B**) T80 cells (grown on coverslips) were treated with 3000 IU/ml IFN-2α (left panel) for 18 hours, transfected with empty pcDNA3, or mock transfected. Cells were immunostained for PLSCR1 (4D2) and DAPI. Alexafluor-488 refers to control cells stained only with secondary antibody (no primary antibody was applied). Images were then captured using an inverted fluorescence microscope (n = 3). (**C**) T80 cells were treated with 3000 IU/ml IFN-2α for 24 hours or transfected with empty pcDNA3 for 48 hours. Cell lysates were analyzed via western blotting with the specified antibodies (n = 3) (left panel). Densitometric analysis of PLSCR1 western was performed using Image J (n = 3) (right panel)

To determine whether induction of PLSCR1 mediated by dsDNA transfection using pcDNA3 could be generalizable to other vectors, we transfected cells with the following plasmids: pGL3-basic (4.8 kb), pBABE-puro (5.2 kb), pQCXIN (7.4 kb), and pcDNA3 (5.4 kb). As presented in Fig. 2A, we observed that all these plasmids markedly increased PLSCR1 protein to similar levels suggesting that PLSCR1 induction is not vector specific or related to the size of the dsDNA. Since previous studies indicate that IFN-2α stimulation leads to plasma membrane localization of *de novo* synthesized PLSCR1 [22,28], we assessed the subcellular localization of PLSCR1 upon dsDNA transfection in T80 cells transfected with pcDNA3. We performed immunostaining of PLSCR1 using the 4D2 mouse monoclonal antibody. Consistent with the western analysis showing a ∼1.8-fold increase in PLSCR1 protein upon dsDNA transfection (48 hours) in T80 cells relative to IFN-2α treatment (24 hours) (Fig. 2C), there was also a dramatic increase in the intensity of PLSCR1 as assessed by immunofluorescence staining in vector-transfected T80 cells (relative to mock) (Fig. 2B, right panel). We noted that, in the majority of the cells, PLSCR1 was localized primarily to the plasma membrane; however, PLSCR1 could also be detected in the perinuclear regions (i.e. endoplasmic reticulum/Golgi). Although IFN-2α treatment led to plasma membrane localized PLSCR1, the intensity of its expression was markedly lower (Fig. 2B, left panel). Collectively, these results indicate that the majority of the *de novo* synthesized PLSCR1 induced following dsDNA transfection localizes to the plasma membrane.

### dsDNA transfection leads to transcriptional induction of TLRs and IFNs

**Fig 3:**
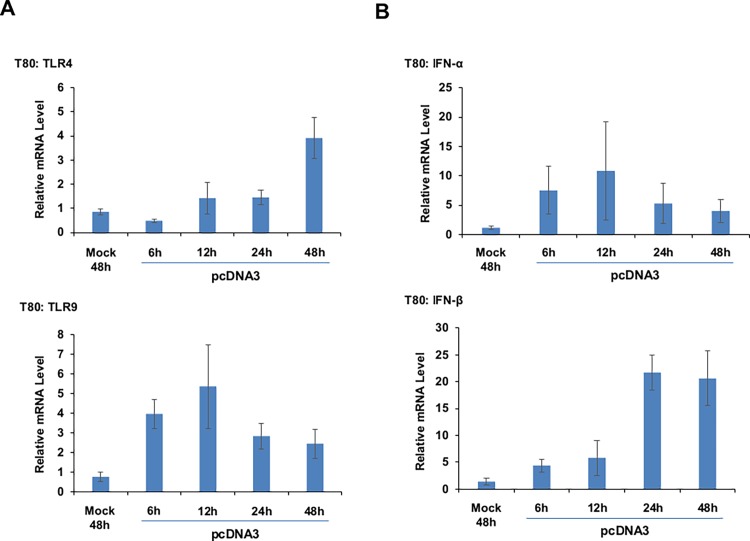
dsDNA transfection leads to induction of TLR and IFN mRNA. T80 cells were mock or empty pcDNA3 transfected. RNA was isolated following 6 to 48 hours transfection. TLR4/TLR9 (**A**) and IFN-α/IFN-β (**B**) mRNA levels were detected by real-time PCR (n = 4).

Toll-like receptors (TLRs) are the major component of the innate immune system and are responsible for recognition of pathogen-associated molecular patterns (PAMPs) including pathogenic foreign nucleic acids (viral and bacterial DNA). Upon recognition of PAMPs, TLRs stimulate the downstream signaling cascade which activates transcription factors, NFκB, and IRF3/7, resulting in induction of inflammatory cytokines including Type 1 IFNs [3]. In addition, the cGAS-STING pathway can upregulate IFNs transcription via activation of IRF3 [12,13]. Since we have observed that IRF3 is phosphorylated following dsDNA transfection (Fig. 1C) and that PLSCR1 can regulate TLR9 signaling in plasmacytoid dendritic cells (pDCs) [21], we next assessed whether TLR4 as well as TLR9 were altered at their transcript level following dsDNA transfection in T80 cells. Both TLR4 and TLR9 have been previously shown to function as nucleic acid sensing TLRs [6]. As expected, there was a significant induction of both TLR4 and TLR9 upon transfection of dsDNA (Fig. 3A, upper and lower panels). Similarly, as shown in Fig. 3B (upper and lower panels), we also detected significant increases in the mRNA levels of IFN-α and IFN-β (IFN-γ was undetectable). Collectively, these results indicate that dsDNA transfection leads to induction of TLR4 and TLR9 as well as IFN-α and IFN-β.

### Inhibition of MAPK does not modulate PLSCR1 mRNA and protein levels upon dsDNA transfection

**Fig 4:**
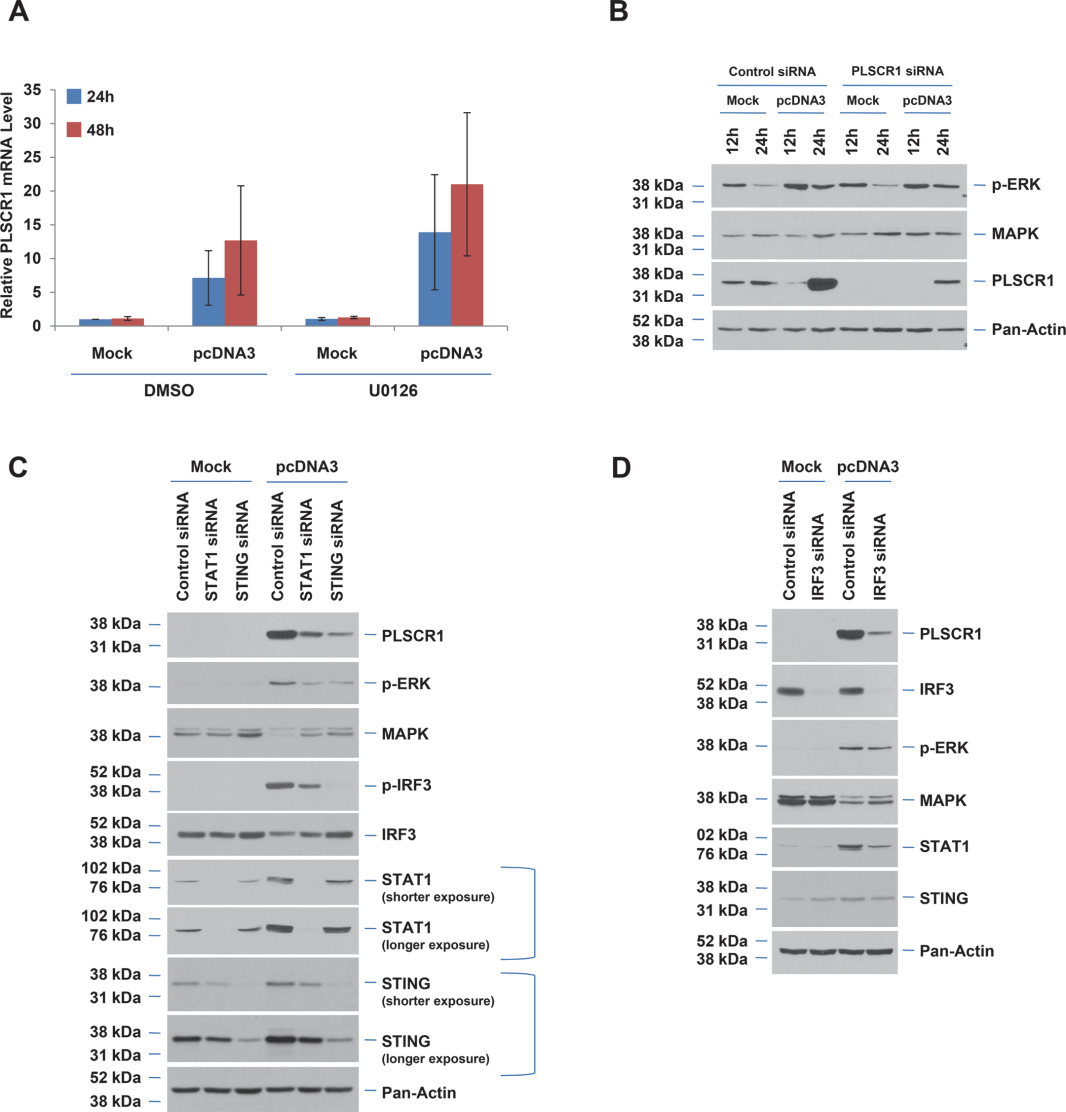
Knockdown of STING, STAT1, or IRF3 leads to reduced PLSCR1 protein. (**A**) T80 cells were pre-treated with 10 μM U0126 or DMSO for at least 2 hours prior to and 6 hours after transfection with pcDNA3. PLSCR1 mRNA levels were quantified by real-time PCR (n = 3). (**B**) Empty plasmid or mock transfection was performed in T80 cells that were treated with PLSCR1 siRNA. Cell lysates were isolated at the indicated time points following transfection and analyzed via western blotting with the indicated antibodies (n = 2). (**C**) T80 cells were transfected with control, STAT1, or STING siRNA followed by mock or pcDNA3 transfection. Cell lysates were isolated 48 hours post-transfection and analyzed via western blotting with the indicated antibodies (n = 3). (**D**) Empty plasmid or mock transfection was performed in T80 cells that were treated with IRF3 siRNA. Cell lysates were isolated at 48 hours following transfection and analyzed via western blotting with the indicated antibodies (n = 2).

Since dsDNA transfection increased the phosphorylation of MAPK, we then assessed whether MAPK activation contributes to the dsDNA-stimulated PLSCR1 induction. Thus, we pretreated the T80 cells with U0126, an inhibitor of MAP kinase followed by pcDNA3 transfection. However, analysis of the PLSCR1 RNA and protein levels at 24 and 48 hours post-transfection indicated that U0126 did not reproducibly alter PLSCR1 mRNA levels at 48 hours post-dsDNA transfection relative to DMSO treatment (Fig. 4A). Similarly, changes in PLSCR1 protein levels upon U0126 treatment were also variable (Fig. B in S1 File).

Since the activation of MAPK was more sustained upon vector transfection relative to mock transfected cells (up to 48 hours where PLSCR1 levels are also elevated), we wondered whether PLSCR1 may contribute itself to MAPK activation. Thus, to address this question, we performed knockdown of PLSCR1 using siRNA followed by dsDNA transfection for 12 and 24 hours (as shown Fig. 4B). However, PLSCR1 siRNA failed to markedly alter MAPK activation. Together, these results suggest that the MAPK pathway does not contribute to PLSCR1 induction or that PLSCR1 siRNA does not alter MAPK activation upon dsDNA transfection. Since dsDNA transfection also resulted in activation of STAT3 (Fig. 1C), we next assessed whether STAT3 activation is required for the dsDNA-mediated induction of PLSCR1. In this regard, we reduced the expression of STAT3 via siRNA and assessed the changes in the PLSCR1 protein levels. However, we did not observe any significant changes in PLSCR1 protein levels in cells treated with STAT3 siRNA (Fig. C in S1 File) suggesting that PLSCR1 upregulation is independent of STAT3 activation.

### Knockdown of STING or IRF3 ablates the induction of PLSCR1 following dsDNA transfection

In order to assess the involvement of TLR and cGAS-STING signaling pathways in dsDNA-mediated induction of PLSCR1, we attempted to knockdown TLR4 and TLR9, nucleic acid sensing TLRs. However, although we observed changes in TLR4 mRNA upon siRNA treatment, we did not observe any marked changes at the protein level for TLR4. Likewise, we were unable to detect TLR9 mRNA changes upon TLR9 knockdown. Therefore, we were unable to draw conclusions regarding the role of TLR4 and TLR9 in modulating PLSCR1 expression. We next determined whether inhibition of the cGAS-STING pathway could alter the dsDNA-mediated induction of PLSCR1. In this regard, we reduced STING protein levels via siRNA and assessed the dsDNA-induced PLSCR1 protein. We were successful in obtaining efficient knockdown of STING (>90% efficiency at protein level, Fig. 4C). Interestingly, STING knockdown dramatically reduced the PLSCR1 protein and phosphorylated IRF3, a common downstream mediator of cGAS-STING and TLR signaling pathways [12,13,39,41]. In addition, knockdown of IRF3 markedly ablated PLSCR1 induction upon dsDNA transfection with little change in STING protein levels (Fig. 4D). These results indicate that STING may be upstream of IRF3 and PLSCR1 in response to dsDNA transfection. Since we earlier noted that STAT1 protein was markedly elevated following dsDNA transfection (see Fig. 1C) and this molecule had been previously implicated in IFN-mediated PLSCR1 induction [20], we next assessed the effect of STAT1 knockdown using siRNA. As shown in Fig. 4C, STAT1 siRNA led to a partial reduction in STING, p-IRF3, and PLSCR1 protein upon dsDNA transfection. The exact role of STAT1 in this mechanism remains to be understood completely. Collectively, these results indicate that the STING/IRF3 pathway is essential in the upregulation of PLSCR1 expression in the cellular response to dsDNA.

### Lack of PLSCR1 induction in ovarian cancer cell lines

**Fig 5:**
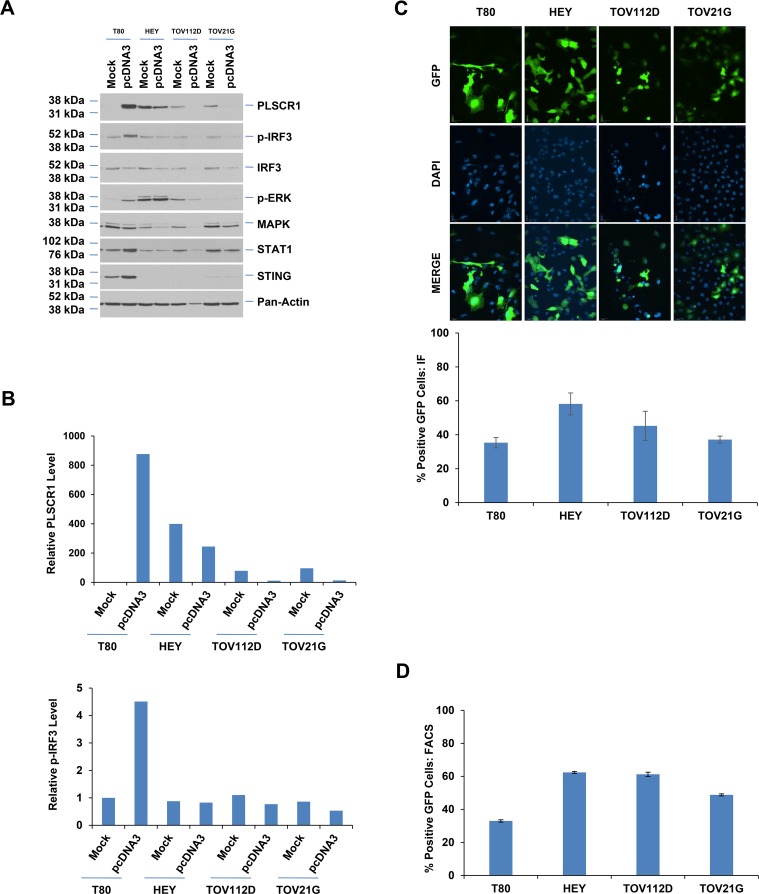
Lack of PLSCR1 induction upon plasmid transfection in ovarian cancer cells. (**A**) T80, HEY, TOV112D, and TOV21G cells were “mock” or empty pcDNA3 transfected. Cell lysates were collected 48 hours post-transfection followed by western analysis with the indicated antibodies (n = 2). (**B**) Densitometric analyses of westerns shown in (**A**) are displayed. (**C**) Transfection efficiency was assessed in T80, HEY, TOV112D, and TOV21G cells (grown on coverslips) using pEGFP-C1 vector. Representative images are presented (top panel). Percentages of GFP positive cells in each of these cell lines is graphically presented (lower panel) (n = 2). (**D**) Transfection efficiency was assessed in T80, HEY, TOV112D, and TOV21G cells using pEGFP-C1 vector by flow cytometry. The percentage of GFP positive cells in each of these cell lines is graphically presented.

To assess whether the cellular response to dsDNA transfection may be different between normal and ovarian cancer cell lines, we utilized serous (HEY), endometrioid (TOV112D), and clear cell (TOV21G) ovarian carcinoma cell lines followed by pcDNA3 transfection. Following 48 hours post-dsDNA transfection, we noted that, in contrast to T80 cells, the ovarian cancer cell lines did not elicit an increase in PLSCR1 protein but rather there was a subtle reduction in its expression (Fig. 5A). The pattern of PLSCR1 expression was similar to the activation of IRF3. We noticed, however, that the total IRF3 was variable following pcDNA3 transfection particularly in the TOV112D and TOV21G cell lines (Fig. 5A); therefore, since the interpretation of the activation status of IRF3 was unclear, we performed densitometric analyses (Fig. 5B). Normalizing p-IRF3 levels to total IRF3 indicated that there was little change in IRF3 activation in the ovarian cancer cells. Interestingly, we noted an increase in STING and STAT1 levels only in T80 cells and not in ovarian cancer cells upon dsDNA transfection suggesting that the absence of IRF3 activation could be due to an altered upstream signaling involving STING and STAT1. To ascertain whether the changes observed in PLSCR1 expression may have been due to differences in transfection efficiency, we transfected these cell lines with pEGFP-C1 plasmid and assessed the GFP-expressing cell population via microscopy (Fig. 5C) and flow cytometry (Fig. 5D). However, as shown in Fig. 5C (top and bottom panels) and Fig. 5D, there did not appear to be a difference in the transfection efficiency among these cell lines which could account for the observed changes in STING, p-IRF3, and PLSCR1 protein.

**Fig 6:**
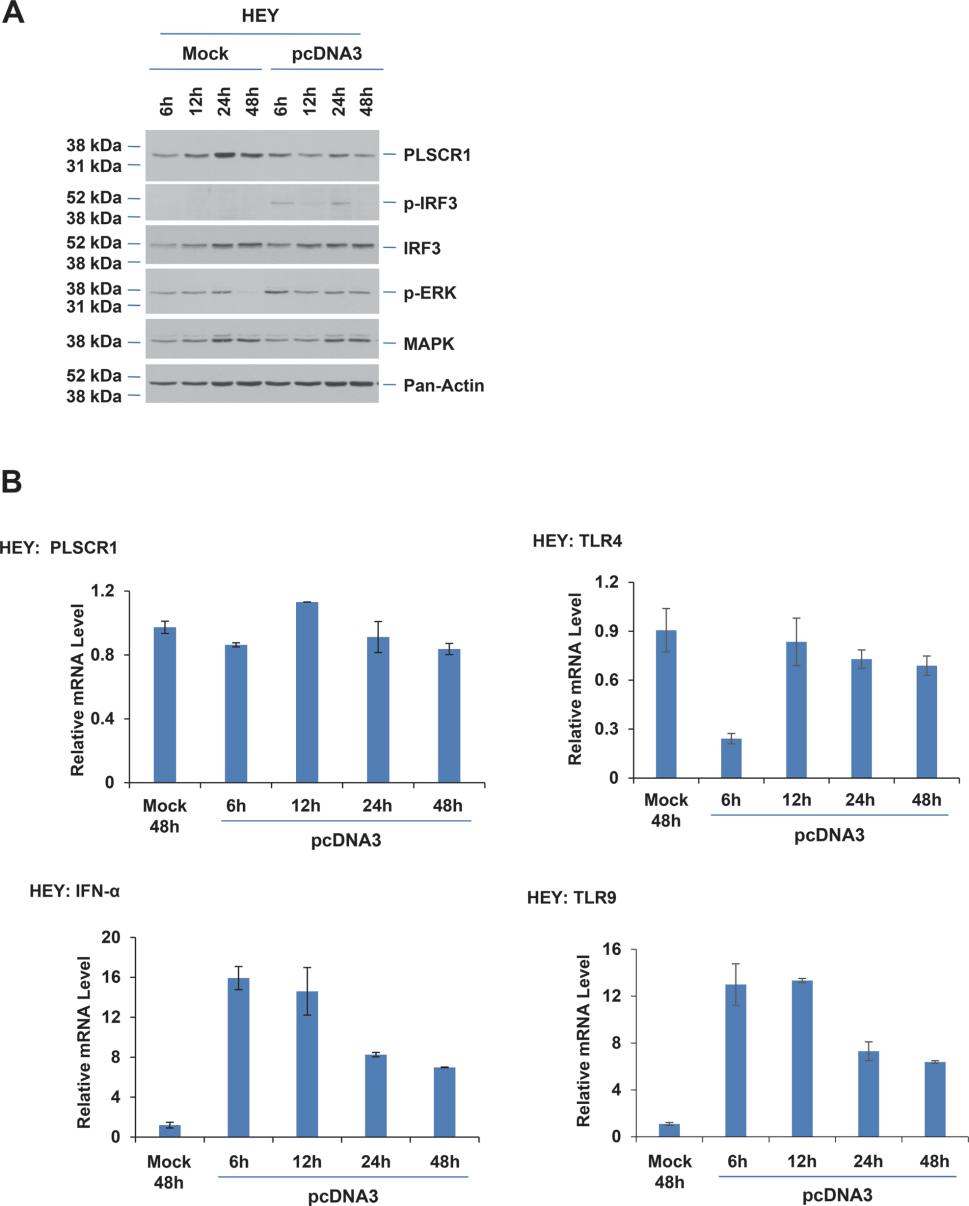
Induction of TLR9 and IFN-α mRNA and a lack of PLSCR1 or TLR4 mRNA in ovarian cancer cells. (**A**) HEY cells were “mock” or pcDNA3 transfected. Cell lysates were collected from 6 up to 48 hours post-transfection and analyzed by western blotting with the indicated antibodies (n = 2). (**B**) Total RNA was isolated from HEY cells that were either mock (48 hours) or pcDNA3 transfected (from 6 up to 48 hours post-transfection). PLSCR1, TLR4, TLR9, and IFN-α mRNA levels were quantified by real-time PCR (n = 2).

To assess whether the kinetics of IRF3 activation and PLSCR1 induction in HEY cells may differ to that of T80 cells, we performed dsDNA transfection from 6 to 48 hours in the HEY cell line. As shown in Fig. 6A, we noted only a slight increase in phosphorylated IRF3 which was variable among the time points assessed and further, we did not identify induction of PLSCR1 at any time points assessed following dsDNA transfection relative to mock. Moreover, as shown in Fig. 6B, TLR4 mRNA was not elevated (as observed for T80 cells (Fig. 3A, upper panel)) although TLR9 mRNA was markedly elevated in HEY cells. Consistent with the protein level changes, PLSCR1 was also not significantly altered at the mRNA level although IFN-α was elevated at 6 hours post-dsDNA transfection. We did not detect IFN-β and IFN-γ mRNA in HEY cells transfected with dsDNA. Whether these changes in HEY, relative to T80 cells, are responsible for the lack of PLSCR1 induction requires further investigation.

## Discussion

**Fig 7:**
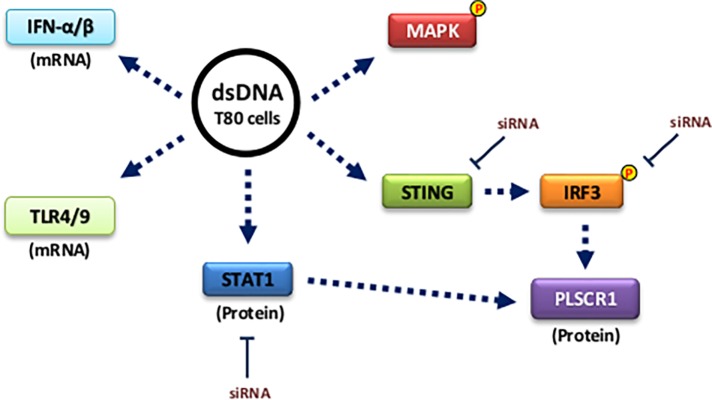
dsDNA transfection in normal epithelial cells induces PLSCR1 expression mediated by IRF3 activation. Plasmid dsDNA transfection in T80 immortalized normal ovarian surface epithelial cells leads to induction of PLSCR1 through the STING/IRF3 pathway. dsDNA transfection also results in activation of MAPK (ERK1/2) and induction of TLR4/9 and IFN-α/β mRNA.

PLSCR1 (a type-II transmembrane protein [42,43]) has previously been implicated in disrupting plasma membrane asymmetry (also attributed to TMEM16F [44,45] and XKR8 [46,47]) and recently to regulate signaling pathways [19,23,48–51]. The PLSCR family is composed of 5 members: PLSCR1, 2, 3, 4 and 5 [52]. It has been reported that growth factors (EGF) and cytokines (IFN) can lead to PLSCR1 upregulation [23,31]. The mechanism by which PLSCR1 is regulated by IFN has been identified to involve PKC-δ, JNK, and STAT1 [20]. Herein, we report a novel finding that transfection with dsDNA in normal immortalized ovarian (T80) and mammary (HMEC) epithelial cells leads to activation of IRF3 and a marked increase in PLSCR1 expression (Fig. 7). We demonstrate that IRF3 knockdown is critical to PLSCR1 induction following dsDNA transfection. In addition, knockdown of STING protein via siRNA decreases activation of IRF3 as well as PLSCR1 protein levels. Existing literature places STING upstream of IRF3 [12,13]. We also noted that STAT1 knockdown could partially decrease PLSCR1 levels. The mechanism by which this occurs requires further investigation. Moreover, the increase observed in IFNα/β mRNA which occurred at 6 hours (shown in Fig. 3B) is unlikely to be due to IRF3 activation since it increased only at 12 hours. Therefore, other pathways such as IRF7 or NFκB activated by the TLRs may contribute to these interferon mRNA changes [53].

Although PLSCR1 is primarily localized to the plasma membrane, studies have also reported it to be localized to various intracellular compartments including the nucleus, endoplasmic reticulum, Golgi, and endosomes [22–24]. Indeed, by site directed mutagenesis of the palmitoylation site of PLSCR1 or cellular treatment with the palmitoylation inhibitor (2-bromopalmitate), PLSCR1 can translocate from the plasma membrane to the nuclear compartment [22]. It is also notable that the cytokine, IFN-2α, can induce plasma membrane or nuclear localization of PLSCR1 in specific cells [22]. The role of PLSCR1 in the nuclear compartment appears to involve transcriptional regulation of inositol 1,4,5-triphosphate receptor (IP_3_R) [54] and interaction with components of the DNA replication machinery including topoisomerases [55]. In our normal ovarian epithelial cells, dsDNA transfection leads to localization of *de novo* synthesized PLSCR1 to the plasma membrane as well as to perinuclear regions (Fig. 2B). Interestingly, PLSCR1 was reported to interact with TLR9 in endosomes [21] which appeared necessary for induction of IFN-α upon CpG-ODN treatment in plasmacytoid dendritic cells [21]. In addition, reduced levels of TLR9 were present in endosomal compartments upon PLSCR1 knockdown [21]. Herein, we demonstrated that the PLSCR1 is modulated via the STING/IRF3 signaling cascade in T80 cells. The function of PLSCR1 at the plasma membrane and endoplasmic reticulum/Golgi upon dsDNA transfection requires further investigation.

There are a number of reports describing the anti-viral activity of PLSCR1. For example, PLSCR1 represses Tat-dependent transactivation of HIV-1 long-terminal repeat as well as *gag* mRNA levels [17]. It can also negatively regulate Tax-mediated transactivation of HTLV-1 LTR [18]. PLSCR1 suppresses hepatitis B and C viral replication which may be mediated through activation of the Jak/STAT signaling cascade [14,15]. Reduced expression of PLSCR1 can also lead to increased levels of vesicular stomatitis as well as encephalomyocarditis viruses [19]. In addition, PLSCR1 expression induced by IFN is essential for cellular defense against the staphylococcus aureus α-toxin [16].

Hepatitis B is a dsDNA virus; interestingly, we noted that HepG2 cells were utilized by Yang *et al*. [14] in their study of HBV infection in which PLSCR1 mRNA was downregulated. However, since HepG2 cells are a hepatocellular carcinoma cell line, it is possible that the mechanism leading to viral-mediated reduction in PLSCR1 expression may be similar to what we observed in our ovarian carcinoma cell lines treated with dsDNA (Fig. 5A). Future studies can be performed to compare and contrast the effect of dsDNA-viral presentation to plasmid dsDNA presentation. Moreover, it would be interesting to determine whether HBV infection in normal cells leads to PLSCR1 induction similar to what we observed in T80 cells.

As presented in Fig. 5A, we noted that PLSCR1 induction was observed only in normal but not in ovarian carcinoma cells. Although the basal levels of PLSCR1 were elevated in the ovarian cancer cell lines relative to the normal T80 cells (Fig. 5A), there was no difference in baseline total IRF3 or phosphorylated IRF3. There exist other IRF family members which may be responsible for regulation of PLSCR1 in the cancer cell lines; certainly, there are reports that various TLRs and IRF family members are expressed differentially in certain cancer cell types [56]. PLSCR1 is located proximal to the 3q26.2 amplicon, which is dysregulated in ovarian as well as other epithelial carcinomas [36]. This region harbors oncogenes including MECOM, SnoN/SkiL, and PKCɩ [36]. Interestingly, we have recently reported that SnoN is partially responsible for upregulating PLSCR1 at the mRNA level [28]. More recently, Snail, an EMT co-transcriptional repressor, has been identified to regulate PLSCR1 transcription [57] potentially implicating a role for PLSCR1 in modulating the aggressiveness of certain cancers. The role of PLSCR1 in inflammation or innate immunity in ovarian cancer development is currently unknown and needs to be further investigated.

## Supporting Information

S1 File Figures A-C. **Figure A. Phosphorylation patterns in RPPA versus RIPA cell lysates**. (**A**) T80 cells were treated with 3000 IU/ml IFN-2α from 15 minutes to 24 hours. Cells were harvested using RPPA or RIPA lysis buffer. Lysates were analyzed by western blotting with the indicated antibodies (n = 2). (**B**) T80 cells were transfected with empty pcDNA3 plasmid (“pcDNA3”) or transfection reagent only (“mock”). Cell lysates were harvested from 6 to 48 hours post-transfection with RPPA or RIPA lysis buffer and analyzed via western blotting with the indicated antibodies (n = 2). **Figure B. Inhibition of MAP kinase activity does not alter PLSCR1 protein upon dsDNA transfection**. T80 cells were pre-treated with 10 μM U0126 for at least 2 hours prior to and after 6 hours post-transfection with empty plasmid. Lysates were collected at 24 and 48 hours post-transfection and analyzed via western blotting with the indicated antibodies (n = 3). Three replicates are presented (1–3). **Figure C. STAT3 knockdown does not alter PLSCR1 induction upon dsDNA transfection**. T80 cells were transfected with siRNA targeting STAT3 followed by empty plasmid pcDNA3 transfection for 24 hours. Cell lysates were then analyzed by western blotting with the indicated antibodies (n = 4). Four replicates are presented (1–4).
(PPTX)
