# Supplementary material for: Induction of PLSCR1 in a STING/IRF3-Dependent Manner upon Vector Transfection in Ovarian Epithelial Cells
Source: PLoS One. 2015 Feb 6;10(2):e0117464. doi: 10.1371/journal.pone.0117464 (PMC4320088; doi:10.1371/journal.pone.0117464)
Supplement: S1 File — Figure A. Phosphorylation patterns in RPPA versus RIPA cell lysates. (A) T80 cells were treated with 3000 IU/ml IFN-2α from 15 minutes to 24 hours. Cells were harvested using RPPA or RIPA lysis buffer. Lysates were analyzed by western blotting with the indicated antibodies (n = 2). (B) T80 cells were transfected with empty pcDNA3 plasmid (“pcDNA3”) or transfection reagent only (“mock”). Cell lysates were harvested from 6 to 48 hours post-transfection with RPPA or RIPA lysis buffer and analyzed via western blotting with the indicated antibodies (n = 2). Figure B. Inhibition of MAP kinase activity does not alter PLSCR1 protein upon dsDNA transfection. T80 cells were pre-treated with 10 μM U0126 for at least 2 hours prior to and after 6 hours post-transfection with empty plasmid. Lysates were collected at 24 and 48 hours post-transfection and analyzed via western blotting with the indicated antibodies (n = 3). Three replicates are presented (1–3). Figure C. STAT3 knockdown does not alter PLSCR1 induction upon dsDNA transfection. T80 cells were transfected with siRNA targeting STAT3 followed by empty plasmid pcDNA3 transfection for 24 hours. Cell lysates were then analyzed by western blotting with the indicated antibodies (n = 4). Four replicates are presented (1–4). (PPTX) [file pone.0117464.s001.pptx]

## Slide 1
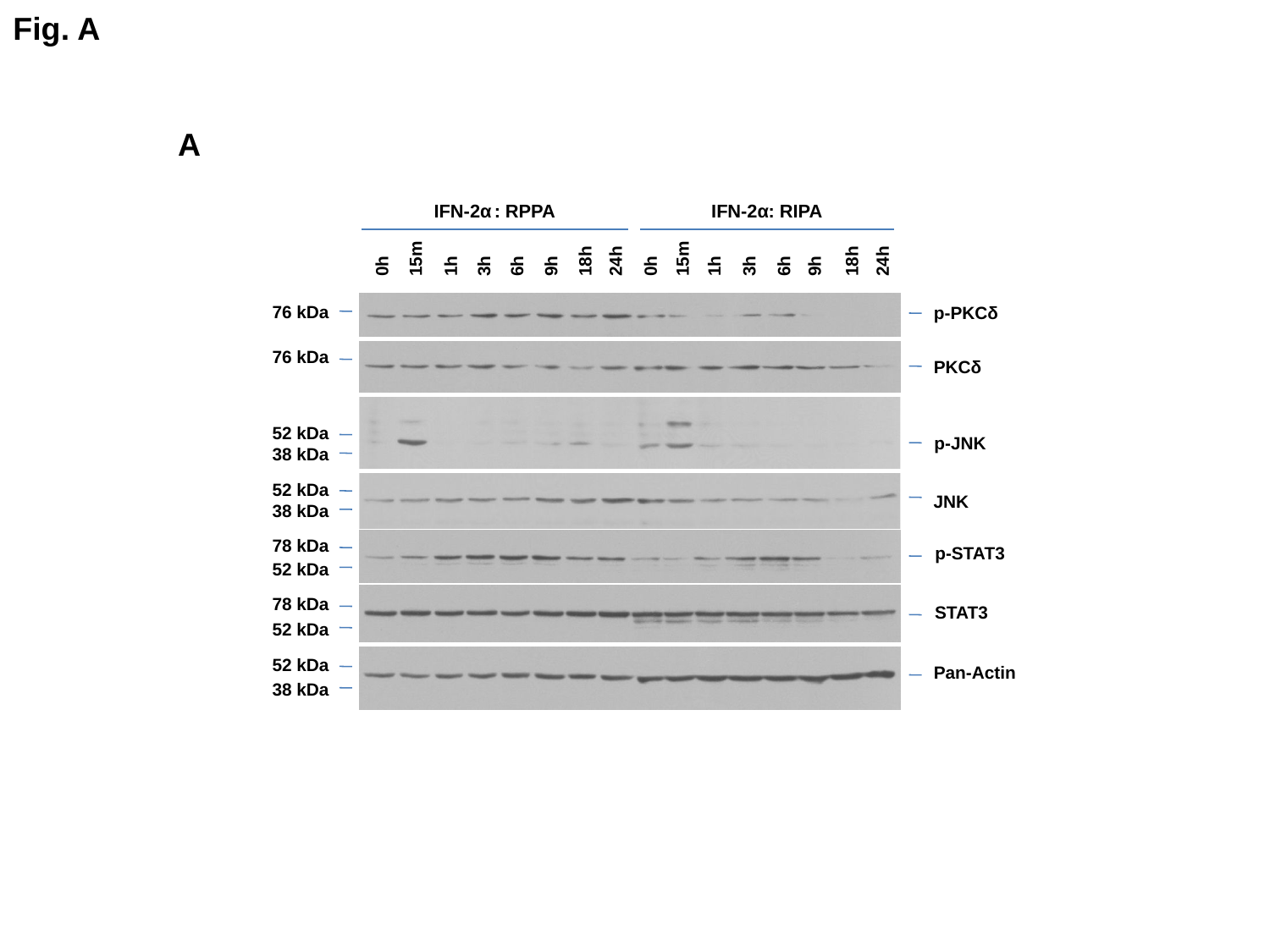

Fig. A
A
IFN-2α : RPPA
IFN-2α: RIPA
3h
6h
9h
18h
24h
3h
6h
9h
18h
24h
0h
15m
1h
0h
15m
1h
76 kDa
p-PKCδ
76 kDa
PKCδ
52 kDa
p-JNK
38 kDa
52 kDa
JNK
38 kDa
78 kDa
p-STAT3
52 kDa
78 kDa
STAT3
52 kDa
52 kDa
Pan-Actin
38 kDa

## Slide 2
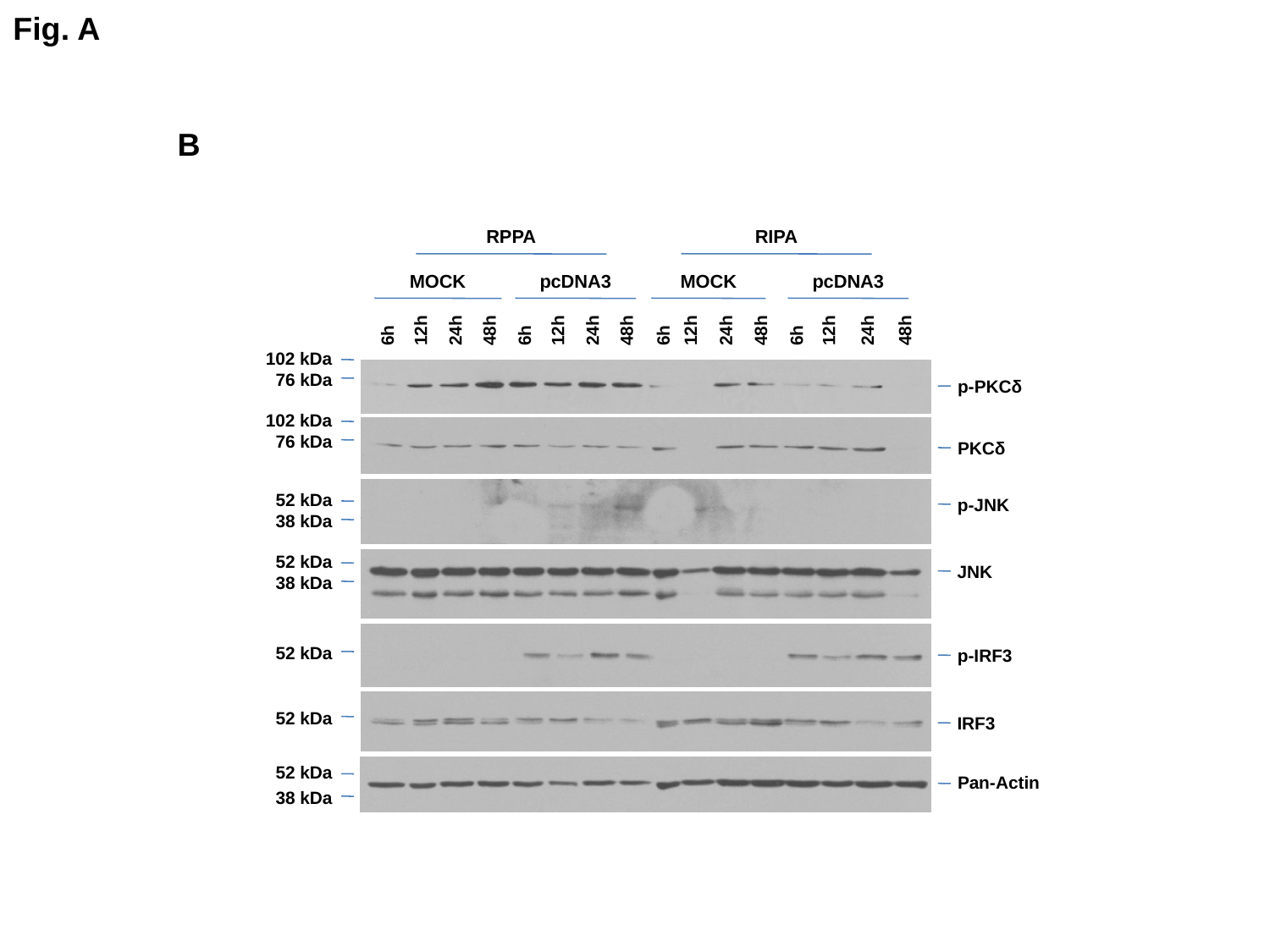

Fig. A
B
RPPA
RIPA
MOCK
pcDNA3
MOCK
pcDNA3
24h
48h
24h
48h
24h
48h
24h
48h
6h
12h
6h
12h
6h
12h
6h
12h
102 kDa
76 kDa
p-PKCδ
102 kDa
76 kDa
PKCδ
52 kDa
p-JNK
38 kDa
52 kDa
JNK
38 kDa
52 kDa
p-IRF3
52 kDa
IRF3
52 kDa
Pan-Actin
38 kDa

## Slide 3
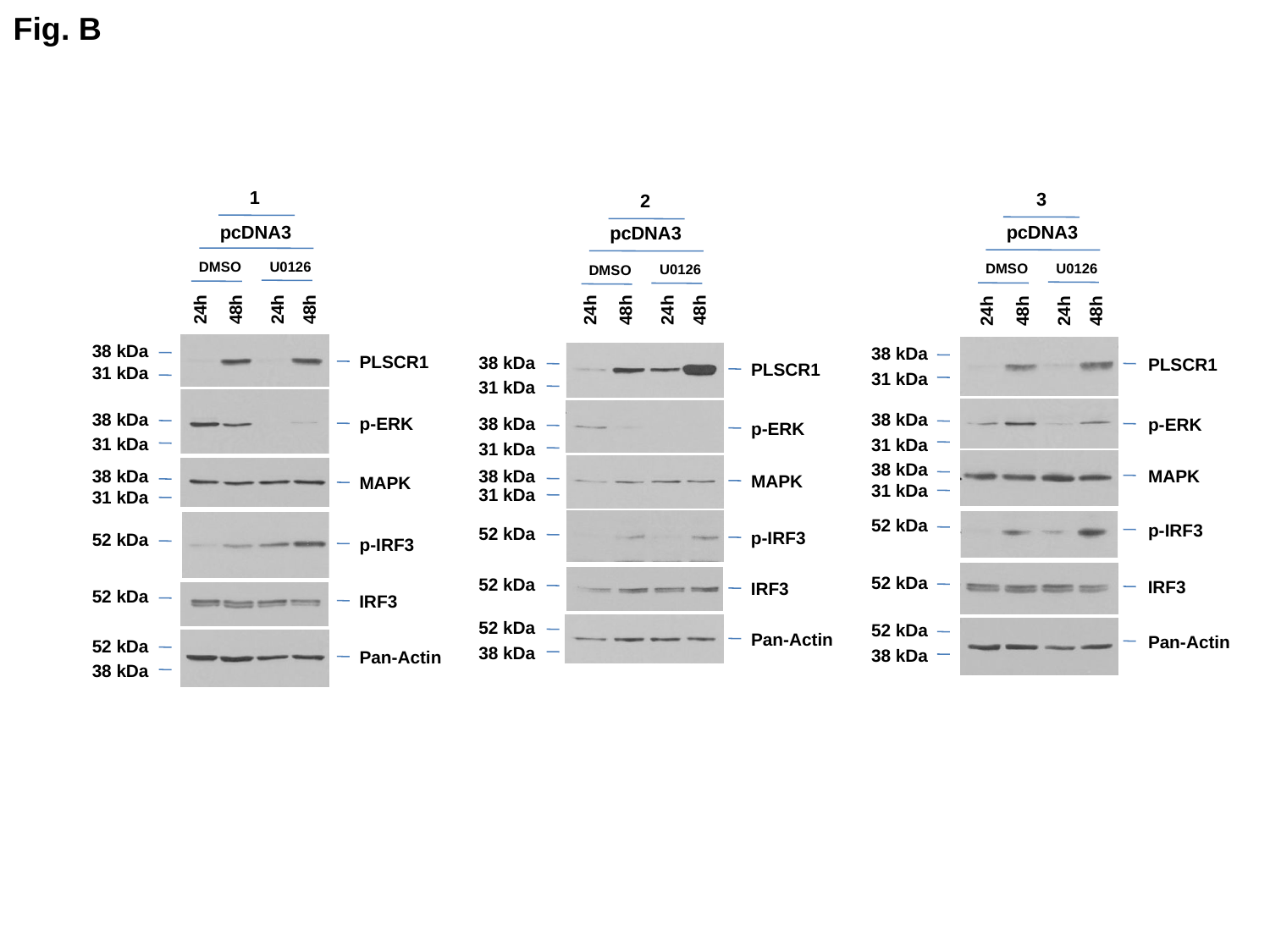

Fig. B
1
3
2
pcDNA3
pcDNA3
pcDNA3
U0126
DMSO
U0126
DMSO
U0126
DMSO
24h
48h
24h
48h
24h
48h
24h
48h
24h
48h
24h
48h
38 kDa
38 kDa
PLSCR1
38 kDa
PLSCR1
PLSCR1
31 kDa
31 kDa
31 kDa
38 kDa
38 kDa
p-ERK
38 kDa
p-ERK
p-ERK
31 kDa
31 kDa
31 kDa
38 kDa
38 kDa
MAPK
38 kDa
MAPK
MAPK
31 kDa
31 kDa
31 kDa
52 kDa
p-IRF3
52 kDa
p-IRF3
52 kDa
p-IRF3
52 kDa
52 kDa
IRF3
IRF3
52 kDa
IRF3
52 kDa
52 kDa
Pan-Actin
Pan-Actin
52 kDa
38 kDa
38 kDa
Pan-Actin
38 kDa

## Slide 4
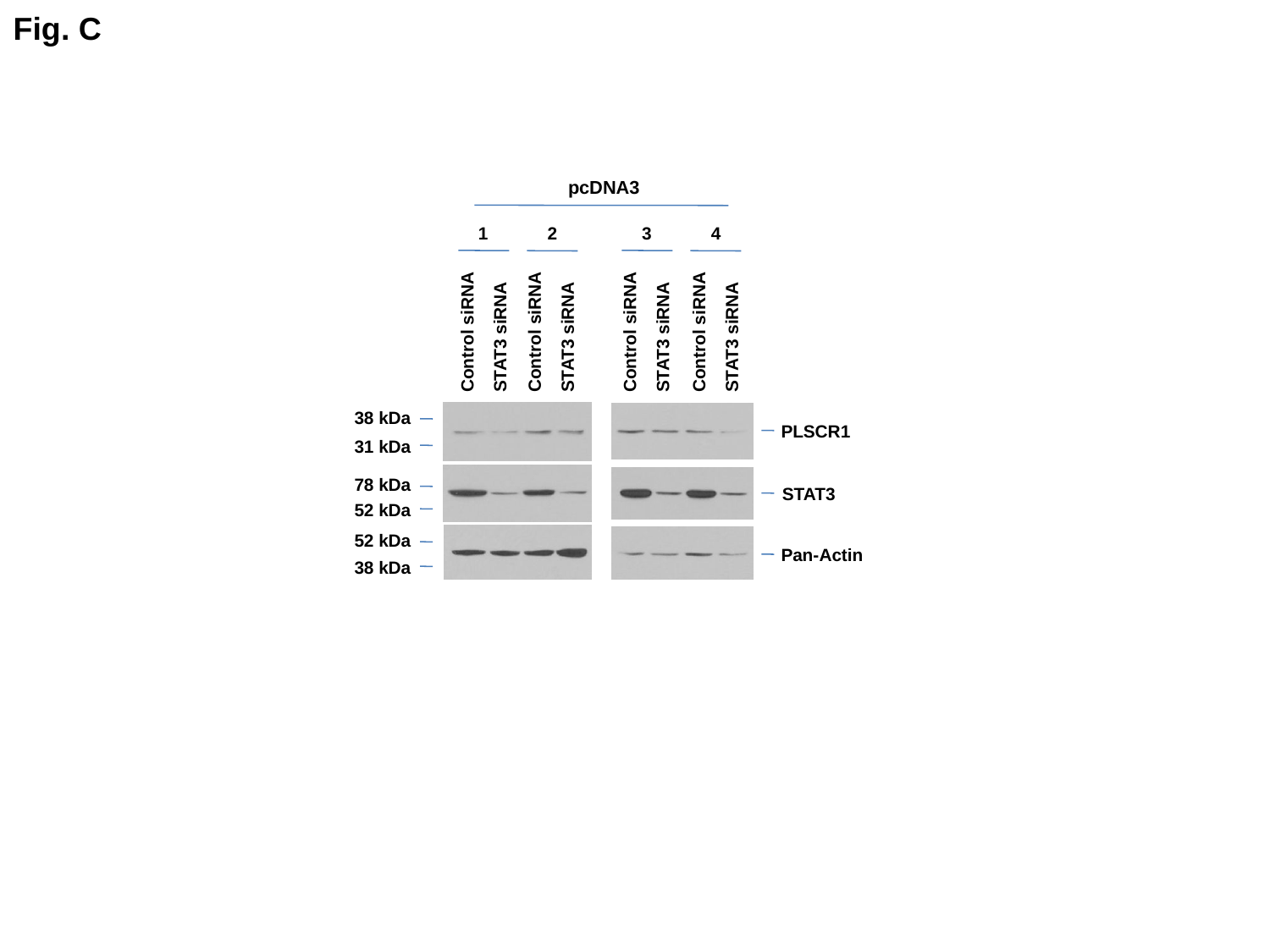

Fig. C
pcDNA3
2
4
1
3
Control siRNA
Control siRNA
Control siRNA
Control siRNA
STAT3 siRNA
STAT3 siRNA
STAT3 siRNA
STAT3 siRNA
38 kDa
PLSCR1
31 kDa
78 kDa
STAT3
52 kDa
52 kDa
Pan-Actin
38 kDa
